# A stepwise antimicrobial and diagnostic stewardship intervention for ordering urine cultures in Saudi Arabia: a quasi-experimental study

**DOI:** 10.3389/fcimb.2026.1684446

**Published:** 2026-05-13

**Authors:** Ahlam Alghamdi, Mohammad Aatif Khan, Mohammed Alraey, Mohammed Alaboud, Isra Farooqi, Asem Allam, Linah Alghamdi, Abdulrahman Alissa, Amani Bahdailah, Mercy Joseph, Thamer A Almangour, Muneerah M Aleissa

**Affiliations:** 1 Department of Pharmacy Practice, College of Pharmacy, Princess Nourah bint Abdulrahman University, Riyadh, Saudi Arabia; 2 Microbiology Laboratory, Department of Pathology and Laboratory Medicine, King Abdullah Bin Abdul-Aziz University Hospital, Princess Nourah bint Abdulrahman University, Riyadh, Saudi Arabia; 3 Department of Infectious Diseases, King Abdullah bin Abdulaziz University Hospital, Riyadh, Saudi Arabia; 4 Pharmaceutical Care Services, King Abdullah bin Abdulaziz University Hospital, Riyadh, Saudi Arabia; 5 Infection Control, King Abdullah bin Abdulaziz University Hospital, Riyadh, Saudi Arabia; 6 Department of Clinical Pharmacy, College of Pharmacy, King Saud University, Riyadh, Saudi Arabia

**Keywords:** antibiotic, antimicrobial resistance, diagnostic stewardship, Saudi Arabia, stepwise, urine culture

## Abstract

**Introduction:**

Inappropriate ordering of urine culture tests can lead to unnecessary antibiotic use and antimicrobial resistance. We evaluated the implementation of a stepwise stewardship intervention, with a focus on the appropriateness of urine culture orders and antibiotic use.

**Methods:**

We conducted a quasi-experimental study from August 2021 to June 2023 at a teaching hospital in Riyadh, Saudi Arabia. We included adult patients with urine culture orders. A stepwise stewardship intervention was implemented in two phases. In the first phase, a clinical decision support (CDS) tool was introduced in February 2022. In the second phase, a reflex urine culture was implemented in January 2023. Our outcomes were the percentage of inappropriate urine cultures ordered and antibiotic use. Outcomes were assessed at baseline, after the CDS intervention, and after the reflex urine culture intervention. We used multivariable logistic regression to estimate the adjusted odds ratio, controlling for potential confounding variables.

**Results:**

During the pre-intervention period, the percentage of inappropriate urine culture orders was 41.6%. Following the implementation of the first intervention—CDS—the percentage decreased to 36.4%. Subsequently, with the addition of reflex urine culture in the second phase of the stepwise intervention, the percentage further decreased to 28.6% (p value=<0.001). The stepwise intervention was associated with 15% lower odds of inappropriate urine culture ordering (adjOR 0.85; 95%CI 0. 0.69 – 0.90; p = 0.027). A significant reduction in unnecessary antibiotics was observed across the three phases: 85.7% pre-intervention, 72.9% post-CDS, and 25% post-CDS and reflex urine culture (p <0.0001). The stepwise intervention was associated with 75% lower odds in unnecessary antibiotic use (aOR 0.25; 95% CI 0.17 – 0.37, p <0.001).

**Conclusions:**

This stepwise intervention suggests that each intervention contributed incrementally to decreasing unnecessary antibiotic use. However, the reduction in inappropriate urine culture request was primarily driven by the implementation of the CDS intervention.

## 1 Introduction

Urinary tract infections (UTIs) are highly prevalent in both the community and healthcare settings (Claeys et al., 2022). Despite being common, appropriate diagnosis and treatment remain a challenge (Claeys et al., 2019). Urine cultures are standard tests ordered for UTIs and they can be the first step that lead to inappropriate antibiotics use, specifically in asymptomatic bacteriuria (ASB), leading to antimicrobial resistance (AMR) (Claeys et al., 2019). International guidelines define urinary tract infections (UTIs) as the presence of a positive urine culture with signs and symptoms and recommend appropriate screening and treatment in such cases (Nicolle et al., 2019; Ministry of Health (KSA), 2020). ASB, on the other hand, is defined as the presence of ≥10^5^ CFU/mL of one or more bacterial species in urine, without UTI signs and symptoms, regardless of pyuria. Current guidelines advise against screening for or treating ASB, except in specific situations such as pregnancy or prior to urological procedures (Nicolle et al., 2019; Ministry of Health (KSA), 2020). The management of ASB in immunocompromised and critically ill patients remain controversial (Nicolle et al., 2019; Ministry of Health (KSA), 2020).

Patients with ASB frequently receive unnecessary antimicrobial therapy (Flokas et al., 2017). This practice is consistent across different healthcare settings and is associated with increased AMR, treatment-emergent adverse effects, and increased healthcare costs (Trautner et al., 2014; Klausing et al., 2016; Spivak et al., 2017). Studies have demonstrated that diagnostic stewardship can work synergistically with antimicrobial stewardship program (ASP) by optimizing appropriate ordering, processing, and reporting of diagnostic tests (Morgan et al., 2017; Claeys et al., 2019; Ostrow et al., 2022; Alghamdi et al., 2025).

In Saudi Arabia, efforts to combat AMR have been implemented as part of the Kingdom’s Vision 2030 Health Sector Transformation Program (Kingdom of Saudi Arabia, 2022). The national AMR action plan — guided by the World Health Organization (WHO) Global Action Plan — supports the development of ASP, automation, and surveillance to optimize antimicrobial use (World Health Organization, 2015; Ministry of Health (KSA), 2020; Ministry of Health (Saudi Arabia), 2024). While ASP has been widely implemented across Saudi Arabia, experience with diagnostic stewardship, specifically for ASB remains limited (Alghamdi et al., 2021; Alshehri et al., 2025). Our center previously implemented and reported its experience with diagnostic stewardship initiatives for urine cultures, which aim to improve patients’ outcomes specifically in ASB. Our first initiative included a clinical decision support (CDS) tool implemented in the electronic health record (EHR). This initiative was associated with a significant reduction in inappropriate urine cultures and unnecessary antimicrobial utilization. The CDS intervention was associated with approximately 17% lower odds of inappropriate urine cultures and 52% lower likelihood of unnecessary antibiotic use (Alghamdi et al., 2025). Although the intervention was successful, a substantial proportion of inappropriate practices persisted, indicating room for further improvement (Alghamdi et al., 2025). As a result, our stewardship team decided to expand the initiative to a reflex urine culture, where the microbiology laboratory evaluates the urinalysis results before proceeding to a urine culture test. In the current study, we sought to evaluate an additional urine reflex diagnostic stewardship initiative on reducing inappropriate urine cultures and unnecessary antibiotic utilization.

## 2 Methods

We conducted a quasi-experimental study at a 400-bed academic medical center in Riyadh, Saudi Arabia. Our hospital serves as a referral center for women, pediatrics, and adolescent health. We compared clinical practice in a pre-intervention phase (August 2021–January 2022), a post-CDS intervention phase (February 2022–July 2022) and a post-reflex urine culture and CDS intervention phase (January 2023–Jun 2023). We included adult patients (≥ 18 years old) from both inpatient and outpatient settings who had urine culture orders during the study period. We excluded patients on antibiotics prescribed for any infections prior to the urine culture, had urine cultures positive for candiduria and those with incomplete data for key variables such as signs and symptoms of UTI, antibiotic indication, and lab results. Consequently, only patients with a clearly documented diagnosis of either symptomatic UTI or asymptomatic bacteriuria were included in the analysis.

### 2.1 Endpoints and definitions

We assessed the impact of our stepwise stewardship interventions, including CDS and reflex urine culture interventions, by measuring inappropriate urine culture and antibiotic utilization. According to the international guideline and institutional ASP committee, appropriate practice for ordering a urine culture includes documented signs and symptoms of UTI, pregnancy, planned invasive urologic procedures, immunocompromised status, or critical illness (Nicolle et al., 2019). Therefore, we defined inappropriate urine culture testing as the absence of documented clinical indications such as signs and symptoms of UTI, pregnancy, urologic procedures, immunocompromised status, or critical illness. Signs and symptoms of UTI include dysuria, flank pain, suprapubic pain, costovertebral angle tenderness, septic shock, and altered mental status in elderly (≥ 65) patients (Nicolle et al., 2019). We defined inappropriate antibiotic utilization when antibiotics were prescribed for ASB without any of the approved indications, including pregnancy, urological procedure, and immunocompromised or critically ill status based on international and local guidelines (Nicolle et al., 2019; Saudi Ministry of Health, 2021). These indications were applied consistently throughout all phases of the study.

### 2.2 Stepwise diagnostic stewardship interventions

#### 2.2.1 CDS intervention

We implemented the institutional ASP-approved CDS in the EHR on February 1^st^, 2022 as described in our earlier work (Alghamdi et al., 2025). This initiative aims to voluntarily guide physicians to appropriately request urine culture.

#### 2.2.2 Reflex urine culture intervention

We implemented the institutional ASP-approved reflex urine culture stewardship initiative in January 2023. The goal of this initiative is to guide the microbiology laboratory to process urine samples received in the laboratory for urine cultures. As per laboratory-developed criteria, all urine samples are first processed for urinalysis and are only set up for urine cultures if at least one of the following criteria is positive in the urinalysis results. These are: presence of white blood cells or bacteria found on microscopic analysis; a positive nitrate result in urine chemistry, patients in obstetrics, urology or oncology departments; specimens labelled as patient is pregnant, or undergoing a urological procedure, patients that are immunocompromised, or in ICU. For these patients, testing depended on the physician’s order; if both urinalysis and urine culture were requested, both were performed. For urine specimens that do not meet these criteria, the microbiology laboratory will only perform a urinalysis test and will not proceed with the urine culture test. Such samples are reported as “urine culture not indicated as per urinalysis”.

### 2.3 Preintervention phase

The control period, from February 2022 to July 2022, preceeded the implementation of any diagnostic stewardship interventions. During this time, the physicians requested urine culture orders without any decision-support mechanisms or restrictions.

### 2.4 Data collection

Data were gathered at three intervals: a 6-month preintervention phase, a 6-month post-CDS intervention phase, and a 6-month post-CDS and reflex urine culture interventions phase.

### 2.5 Statistical analysis

We analyzed categorical data using chi-square test, and continuous data using Kruskal-Wallis. We also utilized a multivariable logistic regression model, adjusting for age, sex, diabetes mellitus, and chronic kidney disease, to evaluate the association between our interventions and inappropriate urine culture orders and unnecessary antibiotic utilization. All analyses were conducted in STATA/BE version 18.

### 2.6 Ethics approval

The study obtained approval from the Institutional Review Board for Ethics in King Abdullah bin Abdulaziz University Hospital (Approval Code: 25-0122). There was no informed consent obtained due to the nature of the study.

## 3 Results

**Figure 1:**
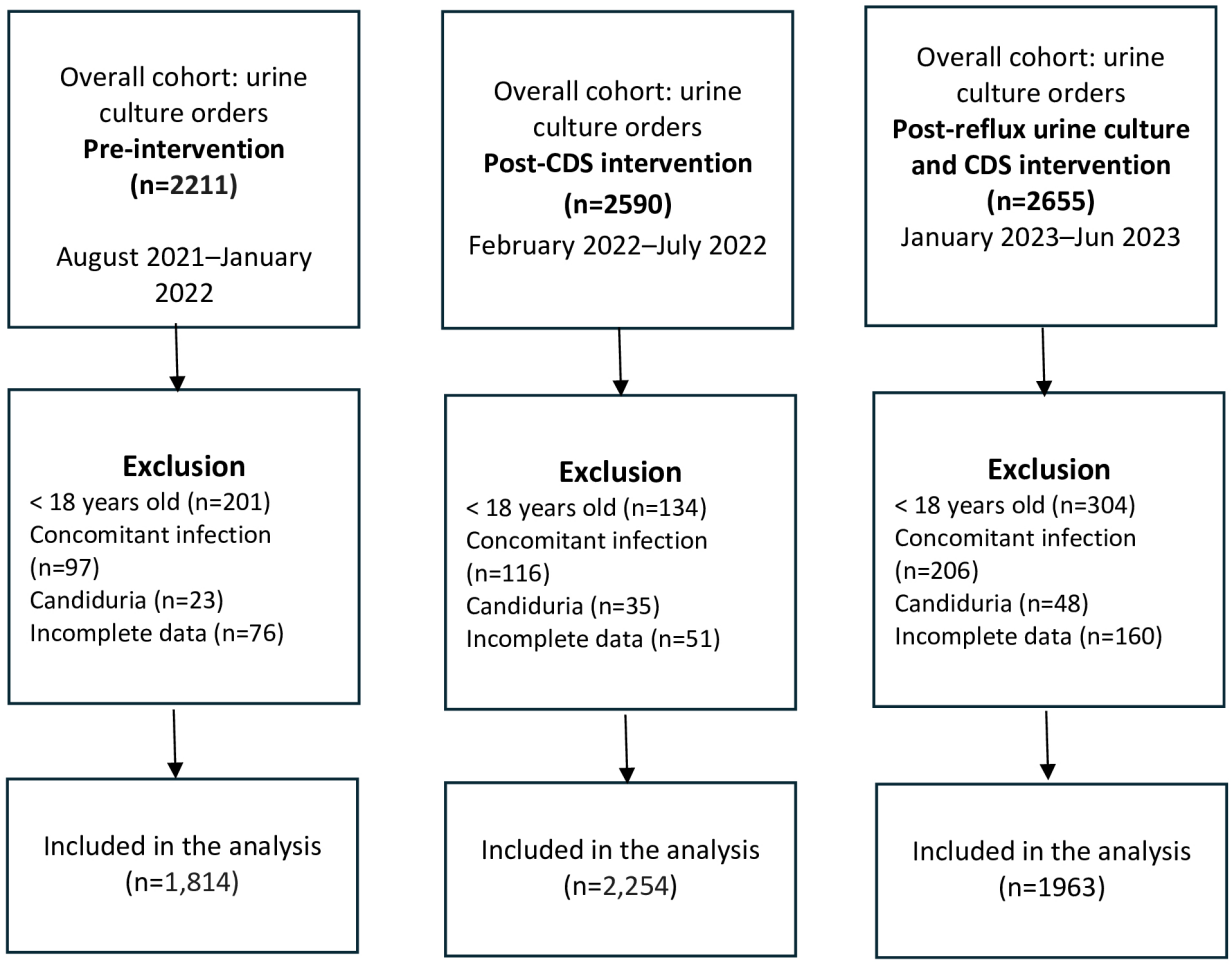
Flow diagram of study participats who had urine culture orders during the study period.

During the study period, we identified 6031 urine culture orders among 5615 unique patients, of which 1814 were ordered in the pre-intervention period, 2254 in the post-CDS intervention period, and 1963 in the post-CDS and reflex urine culture intervention (**Figure 1**). Overall, the mean age was 41 years, and the majority were female (76.1%). Approximately 31.4% of patients were pregnant, 4.9% underwent urologic procedures, 4.7% were immunocompromised, and 2.7% were critically ill. Overall, 11.7% had a positive urine culture (**Table 1**).

**Table 1:** Characteristics of patients who had urine culture orders in post-reflex urine culture intervention, Saudi Arabia.

Number of encounter (N = 6031)	Post-CDS and reflex urine culture intervention (n= 1963)
Patient characteristics	
Age, median [IQR]	38 [29 -53]
Female	1526 (77.7)
Medical Co-morbidities	
Heart disease	251 (12.8)
Diabetes mellitus	454 (23.1)
Hypertension	388 (19.8)
Chronic kidney disease	99 (5)
Liver disease	32 (1.6)
Respiratory disease	199 (10.1)
Active malignancy	24 (1.2)
Pregnancy	596 (30.4)
Urologic procedure	121 (6.2)
Immunosuppressant agent	75 (3.8)
ICU at index culture	89 (4.5)
Urine culture characteristics	
Total number of urine culture performed*	1473 (75)
Positive urine culture	276 (14)

Variables are expressed as n (%) unless indicated otherwise.

ASB, asymptomatic bacteriuria; ICU, intensive care unit; IQR, interquartile range; UTI, urinary tract infection.

*Urine samples were requested for all patients; however, urinalysis and urine culture were not performed in all cases.

Data from the pre-intervention and CDS phase have been previously published (Alghamdi et al., 2025).

During the pre-intervention period, the percentage of inappropriate urine culture orders was 41.6%. Following the implementation of the first intervention—CDS—the percentage decreased by 36.4%. Subsequently, with the addition of reflex urine culture in the second phase of the stepwise intervention, the percentage further decreased by 28.6%. The reduction observed across the three periods was statistically significant (p < 0.001) (**Table 2**).

**Table 2:** Inappropriate urine culture orders and unnecessary antibiotic use in pre- and post-implementation of CDS and microbiology initiatives.

Variable	Pre-intervention	Post-CDS intervention	Post-CDS and reflex urine culture intervention	P value
Number of inappropriate urine culture requests, n (%)	754 (41.6)	821 (36.4)	562 (28.6)	<0.001
Number of unnecessary antibiotic use, n (%)	288 (85.7)	310 (72.9)	68 (25)	<0.001

Total number of prescribed antibiotics were 336, 761 and 272 in pre-, post-CDS implementation, and post- CDS and reflex urine culture intervention period.

Total number of requested cultures were 1814, 2254 and 1963 in pre-, post-CDS implementation, and post- CDS and reflex urine culture intervention period.

Data from the pre-intervention and post-CDS intervention have been previously published (Alghamdi et al., 2025).

Overall, the stepwise intervention was associated with a 14% lower likelihood in inappropriate urine culture orders (OR 0.86; 95% Cl 0.75 – 0.99; p = 0.047). These results remained significant after adjusting for confounders (**Tables 2**, **3**). When evaluating the impact of adding a reflex urine culture intervention to an existing CDS intervention, we found that the combined intervention was not associated with a reduction in inappropriate urine culture orders compared to the CDS intervention alone (OR 1.08; 95% CI 0.94 – 1.23, p = 0.28) (**Table 3**).

**Table 3:** Outcomes of the antimicrobial stewardship initiatives in pre- and post-implementation using logistic regression model.

Variables	OR (95% CI, p-value)	adjOR* (95% CI, p-value)
Inappropriate urine cultures		
Stepwise intervention vs. no intervention	0.86 (0.75 – 0.99, p = 0.047)	0.85 (0.69 – 0.90, p = 0.027)
Stepwise intervention vs. CDS intervention alone	1.08 (0.94 – 1.23, p = 0.28)	1.08 (0.94 – 1.24, p =0.30)
Unnecessary antibiotic use		
Stepwise intervention vs. no intervention	0.26 (0.18 – 0.38, p<0.001)	0.25 (0.17 – 0.37, p <0.001)
Stepwise intervention vs. CDS intervention alone	0.58 (0.42 – 0.80, p=0.001)	0.52 (0.37 – 0.73, p <0.001)

*Adjusted for sex, age, diabetes mellitus, and chronic kidney disease.

Post-CDS intervention results were previously published (Alghamdi et al., 2025).

Additionally, we identified that 1369 antibiotic regimens were prescribed: 336 in the pre-intervention period, 761 in the post-CDS intervention period, and 272 in the combined intervention period. During the pre-intervention period, the percentage of unnecessary antibiotics was 85.7%. Following the implementation of the CDS intervention, based on physician review of symptoms and indications, reduced to 72.9% (Alghamdi et al., 2025). Subsequently, with the addition of reflex urine culture in the second phase of the stepwise intervention, the percentage further decreased to 25%. The reduction observed across the three periods was statistically significant (p < 0.001) (**Table 2**).

Overall, the stepwise intervention was associated with 74% lower odds in unnecessary antibiotic use (OR 0.26; Cl 0.18-0.38; <0.001). Moreover, the addition of a reflex urine culture intervention to the existing CDS-intervention was associated with 48% lower odds in unnecessary antibiotic use compared to CDS intervention alone (OR 0.58; Cl 0.42-0.80; <0.001) (**Tables 2**, **3**). These results remained statistically significant after adjusting for confounders (**Table 3**).

## 4 Discussion

To our knowledge, this is the first study assessing a stepwise stewardship approach on the impact of both inappropriate urine culture orders and unnecessary antibiotic utilization for UTI in the Eastern Mediterranean region. Despite international and local guidelines for UTI management, inappropriate antibiotic use remained common, prompting the need to implement further stewardship intervention. The stepwise intervention, which includes a CDS and reflex urine culture was found to significantly contribute to decreasing inappropriate urine cultures and unnecessary antibiotic use.

Multiple studies indicate that inappropriate antibiotic prescribing for UTIs is still common nationally and internationally, highlighting the need for more focused and strategic stewardship interventions to optimize the use of antibiotics in UTI (Alanazi, 2018; Wang et al., 2020; Jung et al., 2022; Alghamdi, 2025). Globally, various stewardship initiatives have been implemented to reduce unnecessary antibiotic use for ASB. These interventions include: education, reflex urine culture, and selective reporting, which consistently lowered inappropriate urine cultures and antibiotic use, without increasing adverse events (Al-Bizri et al., 2022; Claeys et al., 2022; Morado and Wong, 2022). However, in Saudi Arabia, while a national ASP framework is implemented, there is a gap in targeted interventions specifically aimed at reducing unnecessary antibiotic use for ASB (Alshehri et al., 2025). Our study demonstrated that the percentage of urine culture orders and unnecessary antibiotic use declined significantly across all phases, demonstrating a cumulative effect of the stepwise intervention in reducing both inappropriate urine culture and antibiotic use. The combined CDS and urine reflex intervention significantly reduced unnecessary antibiotic use but did not lead to a reduction in urine culture ordering compared with CDS alone. This may be explained by physicians ordering urine cultures as a precautionary measure, suggesting the need for investigation and targeted education to optimize appropriate urine culture utilization. Notably, pregnancy accounted for approximately one third of the urine cultures, which is consistent with our role as a referral center for women.

These findings align with current evidence showing that bundled, multidisciplinary approaches, including education, decision support, and laboratory stewardship, can successfully reduce inappropriate urine cultures and antibiotic use, ultimately improving patient outcomes and supporting ASP efforts (Adams et al., 2020; Grigoryan et al., 2020; Pham et al., 2020; Morado and Wong, 2022). Despite the observed improvements, there is opportunity for further optimization, as 28.6% of urine orders and 25% of antibiotic prescriptions remain inappropriate. Future work should explore the integration of AI-assisted decision support tools to further tailor urine culture ordering and antibiotic prescribing practices. It is worth noting that this study was conducted during and after the COVID-19 pandemic, which may have influenced our findings. Patient numbers increased across the three phases (n=2211, n=2590, and n=2655), with a slightly lower volume in the pre-intervention phase likely reflecting reduced healthcare utilization during the pandemic. Although prior studies suggest that UTI-related care was not substantially impacted, changes in access and prescribing patterns may still represent a potential confounder (Olamijuwon et al., 2024).

Our study has several limitations. First, as a retrospective analysis of a quality improvement initiative, our findings may be subject to confounding factors. Although we adjusted for multiple variables in our multivariable regression model, the results should be interpreted with caution and ideally validated through controlled, prospective studies. Second, the generalizability of our findings is limited, as our study was conducted at a single center and may not reflect practices in other hospitals in Saudi Arabia and other regions. Third, while assessments of inappropriate antibiotic use were performed by infectious diseases specialists, they were not formally validated, which may have led to discrepancies between evaluators. Finally, the variability in documentation practices, particularly in settings with locum or part-time physicians, could introduce selection bias.

In conclusion, the stepwise intervention indicates that each phase contributed incrementally to the reduction of unnecessary antibiotic use. However, the reduction in inappropriate urine culture request was primarily driven by the implementation of the CDS intervention.

In addition, this project is strongly aligned with both national and international efforts to combat AMR, including Saudi Arabia’s Vision 2030, which prioritizes innovation and healthcare transformation. As the first initiative of its kind in the region, this project serves as a model for future stewardship efforts and highlights the value of collaborative, multifaceted and stepwise interventions in improving antimicrobial use and patient outcomes.

## Data Availability

The raw data supporting the conclusions of this article will be made available by the authors, without undue reservation.

## References

[B1] Adams J. File T. England M. Reynolds N. Wells P. Politis P. (2020). Changing the culture of ordering urine cultures. Infect. Ctrl Hosp. Epidemiol. 41, s162–s162. doi: 10.1017/ice.2020.685. PMID: 41292463

[B2] Alanazi M. Q. (2018). An evaluation of community-acquired urinary tract infection and appropriateness of treatment in an emergency department in Saudi Arabia. Ther. Clin. Risk Manag 14, 2363–2373. doi: 10.2147/TCRM.S178855. PMID: 30584311 PMC6287421

[B3] Al-Bizri L. A. Vahia A. T. Rizvi K. Bardossy A. C. Robinson P. K. Shelters R. T. . (2022). Effect of a urine culture stewardship initiative on urine culture utilization and catheter-associated urinary tract infections in intensive care units. Infect. Ctrl Hosp. Epidemiol. 43, 1032–1035. doi: 10.1017/ice.2021.273. PMID: 34236024

[B4] Alghamdi A. (2025). Evaluating antibiotic prescribing practices for patients with asymptomatic bacteriuria in Saudi Arabia: the need for stewardship initiatives. Inf Dis. Health 13, S2468045125000288. doi: 10.1016/j.idh.2025.04.003. PMID: 40382206

[B5] Alghamdi A. Alkazemi A. Ibrahim A. Alraey M. Alaboud M. Farooqi I. . (2025). Impact of diagnostic stewardship on urine culture ordering in Saudi Arabia: prospective pre- and postintervention study. JMIR Med. Inform. 13, e68044–e68044. doi: 10.2196/68044. PMID: 40700033 PMC12309619

[B6] Alghamdi S. Berrou I. Aslanpour Z. Mutlaq A. Haseeb A. Albanghali M. . (2021). Antimicrobial stewardship programmes in Saudi hospitals: evidence from a national survey. Antibiotics (Basel) 10, 193. doi: 10.3390/antibiotics10020193. PMID: 33671401 PMC7923167

[B7] Alshehri A. Aldali J. Abdelhamid M. Alanazi A. Alhuraiz R. Alanazi L. . (2025). Implementation of antimicrobial stewardship programs in Saudi Arabia: a systematic review. Microorganisms 13, 440. doi: 10.3390/microorganisms13020440. PMID: 40005805 PMC11858812

[B8] Claeys K. C. Blanco N. 0. Morgan D. J. Leekha S. Sullivan K. V. (2019). Advances and challenges in the diagnosis and treatment of urinary tract infections: the need for diagnostic stewardship. Curr. Infect. Dis. Rep. 21, 11. doi: 10.1007/s11908-019-0668-7. PMID: 30834993

[B9] Claeys K. C. Trautner B. W. Leekha S. Coffey K. C. Crnich C. J. Diekema D. J. . (2022). Optimal urine culture diagnostic stewardship practice—results from an expert modified-Delphi procedure. Clin. Infect. Dis. 75, 382–389. doi: 10.1093/cid/ciab987. PMID: 34849637

[B10] Flokas M. E. Andreatos N. Alevizakos M. Kalbasi A. Onur P. Mylonakis E. (2017). Inappropriate management of asymptomatic patients with positive urine cultures: a systematic review and meta-analysis. Open Forum Infect. Dis. 4, ofx207. doi: 10.1093/ofid/ofx207. PMID: 29226170 PMC5714225

[B11] Grigoryan L. Van J. Ramsey D. J. Goebel M. Walder A. Kramer J. . (2020). 92. Successful scale-up of an intervention to decrease unnecessary urine cultures led to improvements in antibiotic use. Open Forum Infect. Dis. 7, S177–S177. doi: 10.1093/ofid/ofaa439.402

[B12] Jung J. Kim B. Kim D. Y. Lee M. S. Park S. Y. Kim T. H. . (2022). 1783. Appropriateness of antibiotics use for patients with asymptomatic bacteriuria or urinary tract infection: a retrospective observational multicenter study in Korea. Open Forum Infect. Dis. 9, ofac492.1413. doi: 10.1093/ofid/ofac492.1413

[B13] Kingdom of Saudi Arabia (2022). Health sector transformation program. Vision 2030. Available online at: https://www.vision2030.gov.sa/en/explore/programs/health-sector-transformation-program

[B14] Klausing B. T. Tillman S. D. Wright P. W. Talbot T. R. (2016). The influence of contaminated urine cultures in inpatient and emergency department settings. Am. J. Inf Ctrl 44, 1166–1167. doi: 10.1016/j.ajic.2016.03.055. PMID: 27311512

[B15] Ministry of Health (KSA) (2020). National action plan for combating antimicrobial resistance 2020–2025 (Riyadh: Ministry of Health).

[B16] Ministry of Health (Saudi Arabia) (2024). Saudi arabia continues national efforts to combat antimicrobial resistance. Saudi Arabia: Jeddah Ministry of Health.

[B17] Morado F. Wong D. W. (2022). Applying diagnostic stewardship to proactively optimize the management of urinary tract infections. Antibiotics 11, 308. doi: 10.3390/antibiotics11030308. PMID: 35326771 PMC8944608

[B18] Morgan D. J. Malani P. Diekema D. J. (2017). Diagnostic stewardship—leveraging the laboratory to improve antimicrobial use. JAMA 318, 607. doi: 10.1001/jama.2017.8531. PMID: 28759678

[B19] Nicolle L. E. Gupta K. Bradley S. F. Colgan R. DeMuri G. P. Drekonja D. . (2019). Clinical practice guideline for the management of asymptomatic bacteriuria: 2019 update by the Infectious Diseases Society of America. Clin. Infect. Dis. 68, 1611–1615. doi: 10.1093/cid/ciz021. PMID: 31506700

[B20] Olamijuwon E. Keenan K. Mushi M. F. Kansiime C. Konje E. T. Kesby M. . (2024). Treatment seeking and antibiotic use for urinary tract infection symptoms in the time of COVID-19 in Tanzania and Uganda. J. Glob. Health 14, 5007. doi: 10.7189/jogh.14.05007. PMID: 38236690 PMC10795859

[B21] Ostrow O. Prodanuk M. Foong Y. Singh V. Morrissey L. Harvey G. . (2022). Decreasing misdiagnoses of urinary tract infections in a pediatric emergency department. Pediatrics 150, e2021055866. doi: 10.1542/peds.2021-055866. PMID: 35773521

[B22] Pham T. H. Huang A. Huang V. Hall S. T. (2020). 172. Evaluation of multifaceted antimicrobial stewardship interventions on the treatment of asymptomatic bacteriuria. Open Forum Infect. Dis. 7, S93–S94. doi: 10.1093/ofid/ofaa439.216

[B23] Saudi Ministry of Health (2021). Urinary tract infection management protocol (Saudi Arabia: Saudi Ministry of Health).

[B24] Spivak E. S. Burk M. Zhang R. Jones M. M. Neuhauser M. M. Goetz M. B. . (2017). Management of bacteriuria in Veterans Affairs hospitals. Clin. Infect. Dis. 65, 910–917. doi: 10.1093/cid/cix474. PMID: 28531289

[B25] Trautner B. W. Petersen N. J. Hysong S. J. Horwitz D. Kelly P. A. Naik A. D. (2014). Overtreatment of asymptomatic bacteriuria: identifying provider barriers to evidence-based care. Am. J. Inf Ctrl 42, 653–658. doi: 10.1016/j.ajic.2014.02.003. PMID: 24713596

[B26] Wang Y. Mitrani-Gold F. S. Xie L. Jaiswal M. Sun X. Joshi A. V. (2020). 123. Treatment patterns and prevalence of inappropriate and suboptimal antibiotic use among females with uncomplicated urinary tract infection in the US. Open Forum Infect. Dis. 7, S190–S191. doi: 10.1093/ofid/ofaa439.433

[B27] World Health Organization (2015). Global action plan on antimicrobial resistance (Geneva: World Health Organization).

